# Histone methyltransferase PRMT6 plays an oncogenic role of in prostate cancer

**DOI:** 10.18632/oncotarget.10061

**Published:** 2016-06-15

**Authors:** Diogo Almeida-Rios, Inês Graça, Filipa Quintela Vieira, João Ramalho-Carvalho, Eva Pereira-Silva, Ana Teresa Martins, Jorge Oliveira, Céline S. Gonçalves, Bruno M. Costa, Rui Henrique, Carmen Jerónimo

**Affiliations:** ^1^ Cancer Biology & Epigenetics Group Research Center, Portuguese Oncology Institute of Porto, Porto, Portugal; ^2^ Department of Pathology, Portuguese Oncology Institute of Porto, Porto, Portugal; ^3^ Department of Urology, Portuguese Oncology Institute of Porto, Porto, Portugal; ^4^ Department of Morphological Sciences, School of Allied Health Sciences (ESTSP), Polytechnic of Porto, Porto, Portugal; ^5^ Life and Health Sciences Research Institute (ICVS), School of Health Sciences, University of Minho, Braga, Portugal; ^6^ ICVS/3B's PT Government Associate Laboratory, Braga/Guimarães, Braga, Portugal; ^7^ Department of Pathology and Molecular Immunology, Institute of Biomedical Sciences Abel Salazar (ICBAS), University of Porto, Porto, Portugal

**Keywords:** androgen receptor, histone methyltransferase, PRMT6, prostate cancer

## Abstract

Prostate cancer (PCa) is a major cause of morbidity and mortality. Until now the specific role of histone methyltransferases (HMTs) deregulated expression/activity in PCa is poorly understood. Herein we aimed to uncover the potential oncogenic role of PRMT6 in prostate carcinogenesis. PRMT6 overexpression was confirmed in PCa, at transcript and protein level. Stable PRMT6 knockdown in PC-3 cells attenuated malignant phenotype, increasing apoptosis and decreasing cell viability, migration and invasion. PRMT6 silencing was associated with decreased H3R2me2a levels and increased MLL and SMYD3 expression. PRMT6 silencing increased p21, p27 and CD44 and decreased MMP-9 expression and was associated with PI3K/AKT/mTOR downregulation and increased AR signaling pathway. In Sh-PRMT6 cells, AR restored expression might re-sensitized cells to androgen deprivation therapy, impacting in clinical management of castration-resistant PCa (CRPC). *PRMT6* plays an oncogenic role in PCa and predicts for more clinically aggressive disease, constituting a potential target for patients with CRPC.

## INTRODUCTION

Prostate cancer (PCa) is the second most common malignant neoplasm diagnosed in men and the sixth leading cause of cancer-related mortality worldwide [1]. Serum PSA screening for PCa remains controversial, leading to significant overdiagnosis and overtreatment [2]. Thus, new diagnostic and prognostic biomarkers are required to improve clinical management and therapeutic decision-making.

Deregulation of epigenetic mechanisms contributes to PCa development and progression [3]. Histone post-translational modifications [PTMs] are involved in gene expression control, influencing chromatin compaction and signaling for several protein complexes. Abnormal expression of histone methyltransferases (HMTs), including Mixed-Lineage Leukemia 2 (MLL2), Mixed-Lineage Leukemia 3 (MLL3), nuclear receptor binding SET domain protein 1 (NSD1) [4], enhancer of zeste homolog 2 (EZH2) [5] or SET and MYND domain containing 3 (SMYD3) [6], was reported in PCa. Recently, in a small cohort of PCa patients, we evaluated the expression of 37 HMTs, including protein arginine methyltransferase 6 (PRMT6), which was overexpressed, eventually discriminating normal from tumorous prostate tissues [6]. PRMT6, a type I arginine methyltransferase, displays high affinity for R2 of H3, exclusively catalyzing H3R2 asymmetric di-methylation [7]. Because H3R2me2 is a repressive mark, PRMT6 activity was associated with transcriptional silencing [7]. The precise functions of PRMT6, however, are not completely understood, although it has been associated with altered cell proliferation, cellular senescence, DNA repair and innate immunity [8-12]. Our main goal was to explore the role of PRMT6 deregulation in prostate carcinogenesis, characterize the putative oncogenic role and its potential clinical impact.

## RESULTS

### *PRMT6* is overexpressed in PCa

**Figure 1:**
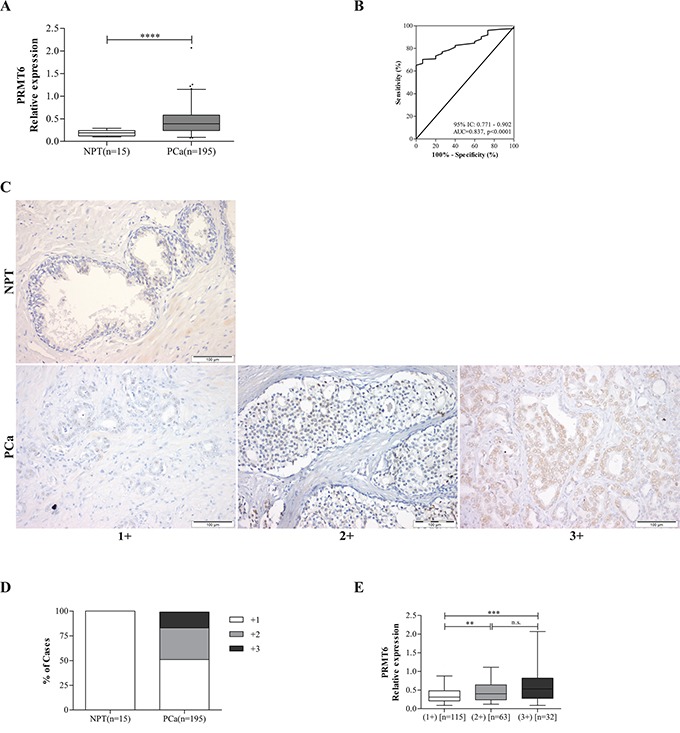
PRMT6 relative expression levels in prostate tissues **A.** *PRMT6* is overexpressed in PCa compared to NPTs; **B.** Performance of *PRMT6* mRNA expression as a biomarker for PCa. ROC curve evaluating the ability of PRMT6 expression levels in discriminating PCa from normal prostate tissues; **C.** Illustrative images of PRMT6 immunostaining in NPT and PCa samples; **D.** Distribution of PRMT6 immunoexpression and of **E.** PRMT6 transcript levels in a series of prostate tissue samples (NPT and PCa), grouped according to PRMT6 immunostaining. ****p<0.0001 (Mann-Whitney U-test); (AUC, area under curve; CI, confidence interval).

Relevant clinical and histopathological data are depicted in Table 1. PCa and NPT age distributions did not significantly differ. PRMT6 was overexpressed in PCa compared to NPT (Figure 1A), but no statistically significant association between PRMT6 expression and standard clinicopathological parameters was found. In ROC curve analysis, using 0.265 expression level as empirical cut-off, PCa tissues were discriminated from NPT with 70.3% sensitivity, 93% specificity, 99.3% positive predictive value (PPV) and 93% negative predictive value (NPV), portraying a global accuracy of 71.9% (Figure 1B).

**Table 1:** Clinical and pathological data of patients

	NPT	PC_A_
***NUMBER OF CASES, N***	15	195
***AGE (Years)***		
Median (range)	64 (45-80)	64 (49-75)
***PSA LEVELS (NG/ML)***		
Median (range)	*N.A.*	8.24 (0-23)
***PATHOLOGICAL STAGE, N (%)***		
pT2	*N.A.*	109 (56%)
pT3a		67 (34%)
pT3b		19 (10%)
***GLEASON SCORE, N (%)***		
< 7	*N.A.*	66 (34%)
= 7		115 (59%)
> 7		14 (7%)

*N.A.*, Not applicable.

Statistically significant increase of PRMT6 immunoexpression from NPTs to PCa (Figure 1C and 1D), as well as association between PRMT6 transcript and protein levels was depicted (Figure 1E) (p<0.001). Statistically significant differences in *PRMT6* transcript levels were detected between immunoscores +1 *vs.* +2 and +1 *vs.* +3 (p<0.01 and p< 0.001, respectively). No association was found, however, between PRMT6 immunoexpression and prognosis.

### PRMT6 is overexpressed and carries prognostic information in TCGA PCa dataset

PRMT6 expression results were validated in a larger and independent dataset, *i.e.*, mRNAseq expression data from PCa patients and matched normal samples deposited in TCGA. PRMT6 expression levels were significantly overexpressed in PCa compared to prostate normal tissues (p<0.0001, Supplementary Figure 1A). Likewise, no significant associations were found between PMRT6 levels and any of the standard clinicopathological parameters. In 52 matched PCa and normal prostate samples, PMRT6 was significantly overexpressed in 89% of the tumors (p<0.0001; Supplementary Figure 1B). Interestingly, higher PRMT6 expression levels were associated with shorter disease-free survival, both in univariate and multivariate analyses (p<0.027) (Supplementary Figure 2).

### Knockdown of PRMT6 in PC-3 and LNCaP cell lines

**Figure 2:**
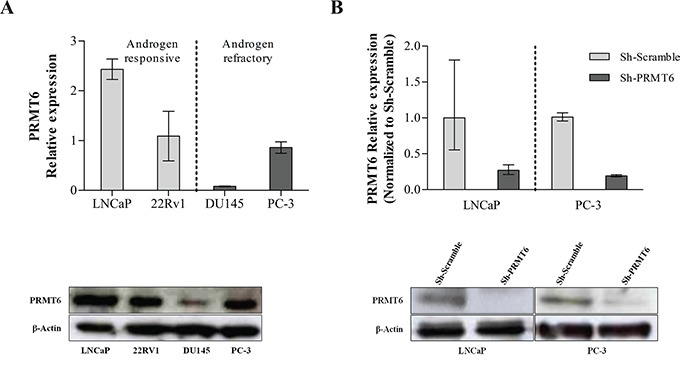
**A.** PRMT6 expression in PCa cell lines at RNA (upper panel) and protein level (lower panel); **B.** The efficiency of PRMT6 knockdown was confirmed at mRNA level, using qRT-PCR (upper panel), and at protein level, using Western-Blot (lower panel), in LNCaP and PC-3 cells. *p<0.05 (Mann-Whitney U-test).

LNCaP, 22RV1, PC-3 and DU145 cell lines expressed PRMT6, at variable levels (Figure 2A). The most highly expressing androgen-responsive and androgen-refractory cell lines (LNCaP and PC-3, respectively) were chosen for subsequent *in vitro* studies. Effective PRMT6 knockdown was achieved in selected cell lines and confirmed at mRNA and protein level (Figure 2B).

### PRMT6 knockdown attenuates malignant phenotype in PC-3 but not LNCaP cells

**Figure 3:**
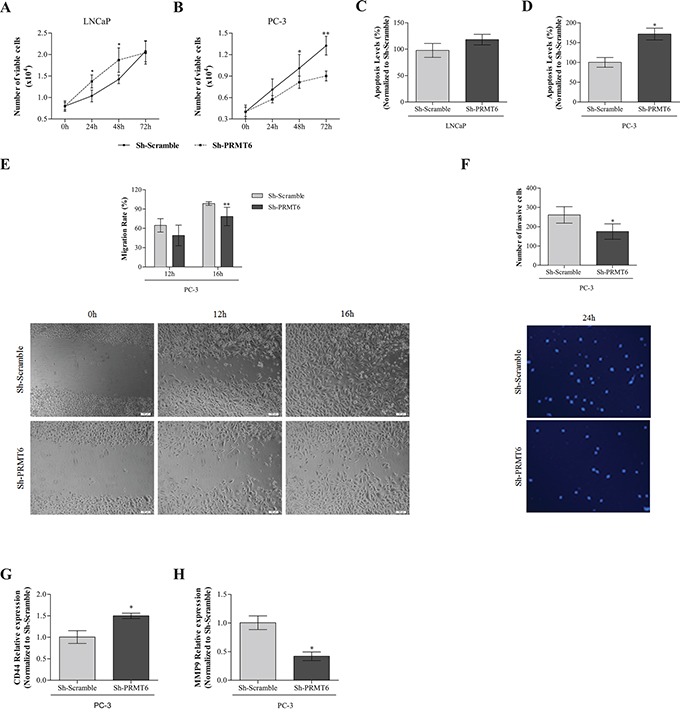
**Impact of PRMT6 knockdown in: A.** LNCaP and **B.** PC-3 cell viability by MTT assay at 0h, 24h, 48h and 72h; **C.** LNCaP and **D.** PC-3 apoptosis by APOPercentage in Sh-Scramble at 72h in Sh-Scramble and Sh-PRMT6; **E.** PC-3 migration by woundhealing scratch assay, representing the upper panel the migration rate at 12h and 16h, and the lower panel illustrative images at the beginning and endpoint of the assay; **F.** PC-3 invasion by matrigel invasion assay, representing the upper panel the percentage of invasive cells at 24h and the lower panel illustrative images at the endpoint of the assay (24h); expression of **G.** *CD44* and **H.** *MMP9* by qRT-PCR in PC-3 Sh-Scramble and Sh-PRMT6. *p<0.05, ***p<0.001 (Mann-Whitney U-test).

Cell viability was evaluated at days 0, 1, 2, and 3. In Sh-PRMT6 LNCaP, increased cell viability at 24h and 48h was observed. However, at 72h, cell viability was similar to Sh-Scramble (Figure 3A). Concerning PC-3, a significant decrease in cell viability was observed after PRMT6 knockdown, at 48h and 72h (Figure 3B). PRMT6 knockdown increased apoptosis, both in LNCaP and PC-3, but only reached statistical significance in the latter (Figure 3C and 3D). A significant decrease in migration ability was observed in Sh-PRMT6 PC-3 (Figure 3E) as well as a decrease in invasion capacity, although it did not reach statistical significance (Figure 3F). These results were confirmed at molecular level, as a significant decrease in *MMP9* and an increase in *CD44* expression was depicted upon PRMT6 silencing (Figure 3G).

### PRMT6 knockdown associates with global H3R2me2a downregulation

**Figure 4:**
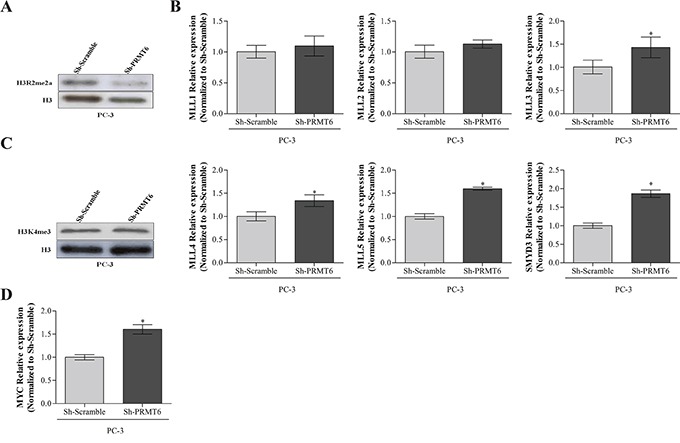
**A.** Reduction of asymmetrical dimethylation of arginine 2 of histone 3 after PRMT6 knockdown in PC-3 cell line; **B.** Relative expression of methyltransferases that catalyze H3K4me3 in PC3 PRMT6-silenced cells namely MLL complex genes and SMYD3; **C.** Western blot analysis of H3K4me3 expression after PRMT6 knockdown in PC-3 cells; **D.** MYC transcript levels in PC-3 PRMT6 silenced cells. *p<0.05 (Mann-Whitney U-test).

Because PRMT6 knockdown induced phenotypic alterations in silenced PC-3 cells, we looked for alterations in specific histone marks it catalyzes [7] and a global H3R2me2a reduction was confirmed (Figure 4A). PRMT6 methyltransferase activity in arginine 2 counteracts MLL complex activity on lysine 4, both in histone 3, which are mutually exclusive [7]. Therefore, we evaluated the impact of PRMT6 knockdown in H3K4 methyltransferases (MLL complex and SMYD3) global expression levels by qRT-PCR, and found that both were significantly increased in PC-3 cells upon PRMT6 knockdown (Figure 4B). The impact of PRMT6 knockdown on MLL complex activity *in vitro* was tested through the evaluation of the differences in H3K4me3 expression in PC-3 cells. However, no significant alterations in expression were apparent (Figure 4C).

Since PRMT6 activity was associated with a decrease in MYC-dependent genes transcription [7] we evaluated MYC transcript levels after PRMT6 silencing. Interestingly, qRT-PCR analysis depicted a significant increase in MYC mRNA levels (Figure 4D).

### PRMT6 knockdown interferes with PCa cell senescence and PI3K/AKT/mTOR pathways

**Figure 5:**
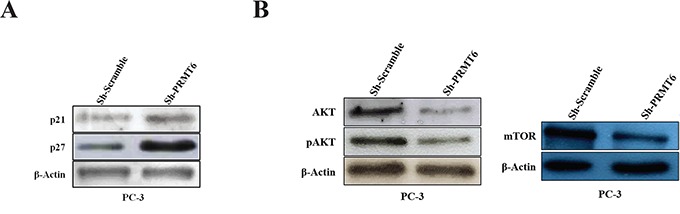
**Western blot analysis of A.** p21 and p27 expression in PC-3 cell line after PRMT6 knockdown; **B.** AKT, pAKT and mTOR expression in PC-3 cell line after PRMT6 knockdown.

Seeking for genes regulated by PRMT6, we evaluated putative targets associated with cellular processes and pathways relevant in PCa. Firstly, cellular senescence was investigated, as this biological process has been previously associated with PRMT6 activity [11, 12]. In PC-3 Sh-PRMT6 cells, western blot analysis showed a substantial increase of p27 expression whilst p21 was only slightly increased (Figure 5A).

The PI3K/AKT/mTOR pathway, which is frequently deregulated in PCa [13] was also investigated. In PC-3 Sh-PRMT6 cells, AKT, pAKT and mTOR protein expression was significantly decreased (Figure 5B), suggesting PI3K/AKT/mTOR pathway downregulation associated with PRMT6 silencing.

### PRMT6 knockdown increases AR expression in PCa cell lines

**Figure 6:**
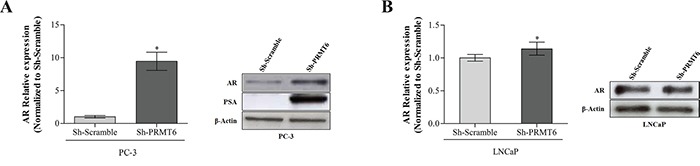
**AR expression in A.** PC-3 PRMT6 silenced cells; qRT-PCR analysis of AR transcript levels (left panel) and western blot analysis of AR and PSA protein levels in PC-3 cell line (right panel) demonstrating that both AR and PSA are overexpressed after PRMT6 silencing; **B.** LNCaP PRMT6 silenced cells both at transcript (left panel) and protein level (right panel). *p<0.05 (Mann-Whitney U-test).

The AR pathway, which is critically involved in PCa initiation and progression, was examined. In PC-3 cells, which have only residual AR expression, PRMT6 knockdown led to AR upregulation, both at transcript and protein level. To confirm these results, PSA (a well-known downstream target of AR) expression was assessed, and an impressive increase in PSA protein levels was apparent in PC-3 Sh-PRMT6 cells (Figure 6A). PRMT6-silenced LNCaP cells displayed a significant increase in AR mRNA but just a slight increase at protein level (Figure 6B).

Moreover, to assess whether PRMT6 is directly related to AR, we stably overexpressed AR in PC-3 cells. AR overexpression in PC-3 cells, at 72h, significantly associated with increased cell viability and decreased apoptosis (Supplementary Figure 3).

## DISCUSSION

Altered HMTs expression patterns have been implicated in prostate carcinogenesis and disease progression [4-6]. Following our previous observation of PRMT6 overexpression in PCa [6], we attempted to confirm its putative oncogenic role and potential clinical implications.

Globally, altered pattern of PRMT6 in PCa mimics that of SMYD3, another HMT which targets different aminoacidic residues [6]. Although no significant associations with standard clinicopathological parameters were depicted, either in our series or TCGA dataset, PRMT6 overexpression associated with shorter interval to biochemical recurrence, suggesting that it imparts a more aggressive phenotype. Interestingly, PRMT6 transcript and protein levels were globally concordant, although no significant differences were depicted between scores +2 and +3. This is most likely due to the semi-quantitative nature of immunohistochemical scoring, which may not be able to discriminate among subtle, yet biologically significant, differences in protein levels. Indeed, mRNA quantitation is more objective and may better discriminate tumors with different biological and clinical aggressiveness, as demonstrated for SMYD3 [6].

Whereas an attenuation of the malignant phenotype (decreased cell viability and increased apoptosis) was consistently observed in Sh-PRMT6 PC-3, no consistent effect was apparent for LNCaP. This might be related to AR regulating role of PRMT6. Because PRMT6 activity leads to AR downregulation, its silencing increases AR transcription. Thus, it is tempting to speculate whether Sh-PRMT6 LNCaP would have survival advantage (as verified at 72h) due to increased AR availability. Sh-PRMT6 PC-3 also demonstrated impaired migration capacity, that was validated at molecular level through *MMP-9* (which promotes neoplastic cell invasion and metastasis [14]) downregulation and *CD44* (involved in cell signaling and cell-cell and cell-matrix adhesion [15]) upregulation. Decreased CD44 expression was reported in primary and metastatic PCa [16]. Moreover, CD44 downregulation has been associated with increased grade and pathological stage, predicting tumor relapse and biochemical recurrence [17]. Interestingly, decreased CD44 expression in PCa has been associated with aberrant promoter methylation [18].

As expected, decreased H3R2m2 levels upon *PRMT6* knockdown were experimentally verified. Moreover, increased MLL complex expression was observed, although no significant differences in H3K4me3 (the histone mark catalyzed by MLL) levels were apparent, even with concurrent SMYD3 increased expression. Because several other enzymes (which we did not assess) also catalyze the H3K4me3 mark, it is plausible that variations in MLL and SMYD3 might not impact in H3K4me3 global levels, although gene-specific effects of those variations cannot be dismissed.

Surprisingly, PRMT6 silencing caused a significant increase in MYC mRNA levels. It should be recalled that PRMT6-mediated H3R2 dimethylation antagonizes the function of the MLL1 complex [7]. Accordingly, we showed that PRMT6 knockdown in PCa was associated with increased MLLs transcript levels. Importantly, PRMT6 can bind to a subset of MYC-dependent genes and catalyze dimethylation of R2 in histone H3 at the 5′end of target genes. Additionally, PRMT6 leads to transcriptional repression by inhibition of H3K4 trimethylation, whereas PRMT6 detachment from its target genes allows for H3K4 trimethylation, entailing target genes' active transcription [7]. Because MYC is a putative target gene of PRMT6, increased MYC expression is then to be expected following PRMT6 silencing.

The observed increase of p21 and p27 expression levels in Sh-PRMT6 PC-3 cells is in line with previous observations [10, 11], implying that PRMT6 overexpression likely interferes with normal cellular senescence in PCa, facilitating neoplastic transformation. Remarkably, p27 plays an important role in prostate carcinogenesis as its expression decreases with increased tumor grade and stage, and p27 downregulation associates with poor prognosis [19]. Furthermore, a decrease in cell proliferation accompanied by increased cell death due to apoptosis upon induction of p27 expression has been reported in PC-3 cells [20], corroborating our phenotypic assays results.

Alterations in PI3K/AKT/mTOR pathway were reported in 42% and 100% of primary and metastatic PCa, respectively [21], which is in line with downregulation of AKT, phospho-AKT and mTOR upon PRMT6 silencing. Although phosphatases and tensin homolog (PTEN), a negative regulator of PI3K/AKT/mTOR pathway, loss of expression in PCa is a common explanation for increased activity of that signaling pathway [22, 23], PRMT6 might further contribute to PI3K/AKT/mTOR activation. Importantly, both PTEN loss and AKT activation have been associated with biochemical recurrence after radical prostatectomy [24, 25], as well as resistance to radiation [26] and chemotherapy [27].

AR signaling is critical for normal development, function and homeostasis of the prostate and its deregulation has been implicated in tumor initiation and progression [28]. Although primary PCa is mostly an androgen-responsive tumor, the castration-resistant phenotype (CRPC) eventually emerges [28], and several mechanisms have been implicated, including heterogeneous loss of AR expression and aberrant promoter methylation [29]. Additionally, AR loss of expression associates with increased invasiveness [30]. PC-3 cells express AR at very low levels and might be, thus, considered a good model of CRPC. Remarkably, we found that PRMT6 silencing in PC-3 cells was associated with functionally restored AR expression, demonstrated by increased PSA expression. Moreover, AR upregulation might also contribute to decreased migration documented in Sh-PRMT6 PC-3 cells. Interestingly, strategies that restore AR expression and, thus, overcome CRPC, might be of clinical utility. However, AR induced expression in PC-3 cells did not show the same effect in viability and apoptosis as PMRT6 knockdown. This might be due to the previously reported opposed roles of AR expression in PCa cell lines [31]. Nevertheless, PRMT6 downregulation and/or inhibition might provide an innovative therapeutic strategy for CRPC, through restoration of AR expression, re-sensitizing neoplastic cells to androgen-deprivation therapy (ADT).

In summary, PRMT6 overexpression, at transcript and protein level, associated with worse disease-free survival in PCa, is suggestive of an oncogenic role. Stable PRMT6 knockdown attenuates the malignant phenotype in PC-3 but not LNCaP, probably due different baseline AR expression. At molecular level, PRMT6 silencing associates with decreased H3R2me2a levels and increased MLL complex and SMYD3 expression, and affects several key genes/molecules, involved in critical cellular pathways. Finally, restored AR expression in Sh-PRMT6 PC-3 is of potential clinical relevance, suggesting that PRMT6 inhibition may re-sensitize androgen-insensitive neoplastic cells to ADT, providing a novel approach to CRPC management.

## MATERIALS AND METHODS

### Patients and samples

PCa samples (n=195) from patients diagnosed and primarily treated with radical prostatectomy at Portuguese Oncology Institute – Porto, Portugal were prospectively collected. Fifteen normal prostate tissue (NPT) samples, of peripheral zone of prostates without PCa, from patients submitted to radical cystoprostatectomy for bladder cancer, served as controls. All specimens, promptly frozen at -80ºC, were cut for nucleic acid extraction. For routine histopathological examination, formalin-fixed and paraffin-embedded (FFPE) fragments were also collected. Relevant clinical data was retrieved from clinical charts. This study was approved by the institutional review board (IRB-CES-IPOFG-EPE 215/013).

### Immunohistochemistry

PRMT6 expression was assessed by immunohistochemistry in FFPE sections using the anti-PRMT6 mouse monoclonal antibody (Santa Cruz Biotechnology Inc., Santa Cruz, CA) at 1:150 dilution with Novolink™ Polymer Detection System (Leica Biosystems, Germany). Normal testis served as positive control. PRMT6 immunoexpression was scored as +1 (weak expression ≤ 50% of cells), +2 (weak expression > 50% of cells or moderate expression ≤ 50% of cells), +3 (moderate expression in > 50% of cells or intense expression, typically >50% of cells).

### TCGA data in prostate cancer patients

Data on PRMT6 mRNA expression and clinical information (when available) from PCa and matched normal patient samples, deposited in The Cancer Genome Atlas (TCGA) was retrieved. mRNA expression data from samples hybridized at University of North Carolina, Lineberger Comprehensive Cancer Center, using Illumina HiSeq 2000 mRNA Sequencing version 2, were downloaded from TCGA data matrix (http://tcga-data.nci.nih.gov/tcga/tcgaDownload.jsp), including 497 PCa and 52 matched normal samples [32]. To prevent duplicates, when there was more than one portion per patient, median values were used. The provided value was pre-processed and normalized according to “level 3” specifications of TCGA (see https://gdc-portal.nci.nih.gov/ for details). Clinical data of each patient was provided by Biospecimen Core Resources (BCRs). Data is available for download through TCGA data matrix (http://tcga-data.nci.nih.gov/tcga/dataAccessMatrix.htm).

### PCa cell lines

PCa cell lines LNCaP, 22RV1, DU145 and PC-3 were used for *in vitro* studies. LNCaP and 22Rv1 cells were grown in RPMI 1640, whereas DU145 and PC-3 cells were maintained in MEM and 50% RPMI-50% F-12 medium (GIBCO, Invitrogen, Carlsbad, CA, USA), respectively. All basal culture media were supplemented with 10% fetal bovine serum and 1% penicillin/streptomycin (GIBCO, Invitrogen, Carlsbad, CA, USA). Cells were maintained in an incubator at 37ºC with 5% CO2. All cell lines were G-banding karyotyped (for validation) and routinely tested for *Mycoplasma spp*. contamination (PCR Mycoplasma Detection Set, Clontech Laboratories).

### Generation of sh-PRMT6 silenced cell lines

PRMT6 knockdown was achieved through viral transduction, in LNCaP and PC3 cell lines, using MISSION^®^ shRNA Lentiviral Transduction Particles (SHCLNV_TRCN0000299934 and SHCLNV_TRCN0000299933 for LNCaP and PC-3, respectively; Sigma-Aldrich^®^) in the presence of polybrene (Santa Cruz Biotechnology Inc.). Control LNCaP and PC3 were generated using MISSION^®^ Non-Mammalian shRNA Control Transduction Particles (SHC002V; Sigma-Aldrich^®^). After transduction, stable clones with shRNA were selected with Puromycin dihydrochloride (cat. 631306, Clontech Laboratories Inc.) at a final concentration of 3μg/mL or 5μg/mL in LNCaP or PC3 cells, respectively.

### Androgen receptor overexpression

Androgen receptor or control-expressing PC3 cells were generated by cloning AR into the pEZ-Lv105 (Genecopoeia, Rockville, MD, USA). As a control, we used the same vector, but lacking an open reading frame. After confirmation of the insert sequence, Fugene 6 transfection reagent (Promega, Madison, WI, USA) was applied to transfect the plasmid DNA into PC3 cells. Stably-transfected cells were selected using Puromycin (Clontech, Mountain View, CA, USA).

### Real time quantitative PCR (qRT-PCR)

RNA was extracted from cell lines using TRIzol^®^ (Invitrogen, Carlsbad, CA, USA). High-capacity cDNA Reverse Transcription Kit from Applied Biosystems (Foster City, CA, USA) enabled first strand synthesis. Expression of target genes (*PRMT6*, *CD44*, *MMP9*, *AR*, *MLL1*, *MLL2*, *MLL3*, *MLL4*, *MLL5* and *SMYD3*) was quantified using Taqman expression assays (Supplementary Table 1), purchased as pre-developed assays (Applied Biosystems) and normalized to the expression of *GUSB* (housekeeping gene).

### Protein extraction and western blot analysis

Protein was extracted from whole-cell lysates using Kinexus Lysis Buffer with Lysis Buffer Cocktail (Kinexus Bioinformatics Corporation, Vancouver, British Columbia, Canada) and protein concentration was determined using BCA assay (Thermo Scientific, Waltham, MA, USA). Subsequently, 30 μg of total protein were separated by SDS-PAGE, transferred to nitrocellulose membranes and incubated with antibodies against PRMT6, H3R2me2a (1/500 Novus Biologicals, Littleton, CO), H3K4me3, PSA (1/1500 and 1/6000 Abcam, Cambridge, UK), AR, mTOR (1/1000, Cell Signaling Technology, Inc., Danvers, MA), p21, p27 (1/500, BD Biosciences, Franklin Lakes, NJ), AKT (1/500, Santa Cruz Biotechnologies Inc) and pAKT (1/500, Millipore Billerica, MA), as well as histone H3 (dilution: 1:500, Abcam) and beta-actin (dilution: 1:8000, Sigma-Aldrich) as input controls, as appropriate. Blots were developed using Immun-Star™ WesternC™ Kit (BioRad, Hercules, CA). All experiments were performed in triplicate.

### Viability assay

Cell viability was evaluated by MTT assay. Briefly, 4000 and 8000 cells per well from PC-3 and LNCaP were seeded onto 96-well flat bottoned culture plates and allowed to adhere overnight. At each time point 0.5 mg/ml of MTT reagent [3-(4, 5dimethylthiazol-2-yl)-2, 5-diphenyl-tetrazolium bromide] was added to each well, and the plates were incubated in the dark for 2 hours at 37ºC. Formazan crystals were then dissolved in DMSO and absorbance was read at 540 nm in a microplate reader (FLUOstar Omega, BMG Labtech, Offenburg, Germany), subtracting the background, at 630 nm. The number of cells was calculated using the formula: [(OD experiment x Number of cells at day 0) / Mean OD at day 0]. Three replicates were performed for each condition, and at least 3 independent experiments were performed.

### Apoptosis evaluation

Evaluation of apoptosis was performed using APOPercentage apoptosis assay kit (Biocolor Ltd., Belfast, Northern Ireland) according to the manufacturer's instructions. PCa cells were seeded onto 96-well plates at the same concentrations used for MTT assay. Apoptotic cells were assessed upon 72 hours of cell adherence in a FLUOstar Omega microplate reader at 550 nm and the background subtracted at 620 nm. The results were normalized to number of viable cell obtained in the MTT assay according to the following formula (OD of apoptosis assay at day x/OD of MTT at day x). The results of the apoptosis assay on the silenced cells were expressed as the ratio of the values obtained for scramble cells (set as 100%). Three biological independent experiments were performed with methodological triplicates for each experiment.

### Cell invasion assay

Cell invasion was determined using BD BioCoat Matrigel Invasion Chamber (BD Biosciences, Franklin Lakes, NJ, USA). Briefly, 2500 cells in 500 μL of serum-free medium were seeded in Matrigel inserts for 48 hours, after which the non-invading cells were removed with cotton swabs from the upper side of the membrane. The membrane botton containing invading cells was fixed in methanol, washed in PBS and stained with DAPI (Vector Laboratories, Burlingame, CA). All the invading cells were counted under a fluorescent microscope. Three independent experiments were performed for each condition, and at least two experimental replicates were performed.

### Wound-healing assay

Briefly, Sc and Sh PC-3 cells were grown to full confluence in 24-well plates, the medium was removed and scratches were performed using a 100μL tip. Cells were then washed with PBS and medium replaced. Scratch closure was analyzed under the inverted microscope and images were captured at different time points. The calculations were made according to the formula [(S Time zero-S Time point/S Time zero) x 100, where S = Distance)]. Three biological independent experiments were performed with methodological quadruplicates for each experiment.

### Statistical analysis

Multiple and pairwise comparisons were performed using the Kruskal-Wallis and Mann-Whitney U tests, respectively, both in clinical samples and in *in vitro* experiments. A receiver operator characteristic (ROC) curve was constructed to assess the performance of PRMT6 as diagnostic biomarker. Spearman correlation tested association between transcript levels of different genes. Fisher's exact test measured association between prostate sample type and PRMT6 immunoexpression.

Survival analysis was performed in TCGA dataset. Prognostic significance of available clinical parameters (pathological stage, GS, age, and serum PSA levels) was assessed by constructing disease-specific and disease-free survival curves using the Kaplan-Meier method with log-rank test (univariate analysis). A Cox-regression model comprising the four variables (multivariate analysis) was also constructed. Disease-free survival was calculated from the date of radical prostatectomy to date of biochemical recurrence, or date of last follow-up or death if recurrence-free. For survival analyses purposes, all cases were coded according to median PMRT6 transcript and serum PSA levels.

Statistical significance was set at P<0.05. Bonferroni's correction was applied to pairwise comparisons. Statistical analyses were performed using SPSS software, version 22.0 (IBM-SPSS Inc., Chicago, IL, USA).

## SUPPLEMENTARY MATERIALS FIGURES AND TABLE


