# Supplementary material for: Histone methyltransferase PRMT6 plays an oncogenic role of in prostate cancer
Source: Oncotarget. 2016 Jun 15;7(33):53018–28. doi: 10.18632/oncotarget.10061 (PMC5288165; doi:10.18632/oncotarget.10061)
Supplement: Supplementary file 1 [file oncotarget-07-53018-s001.pdf]

Histone methyltransferase PRMT6 plays an oncogenic role of in prostate cancer

SUPPLEMENTARY FIGURES AND TABLE

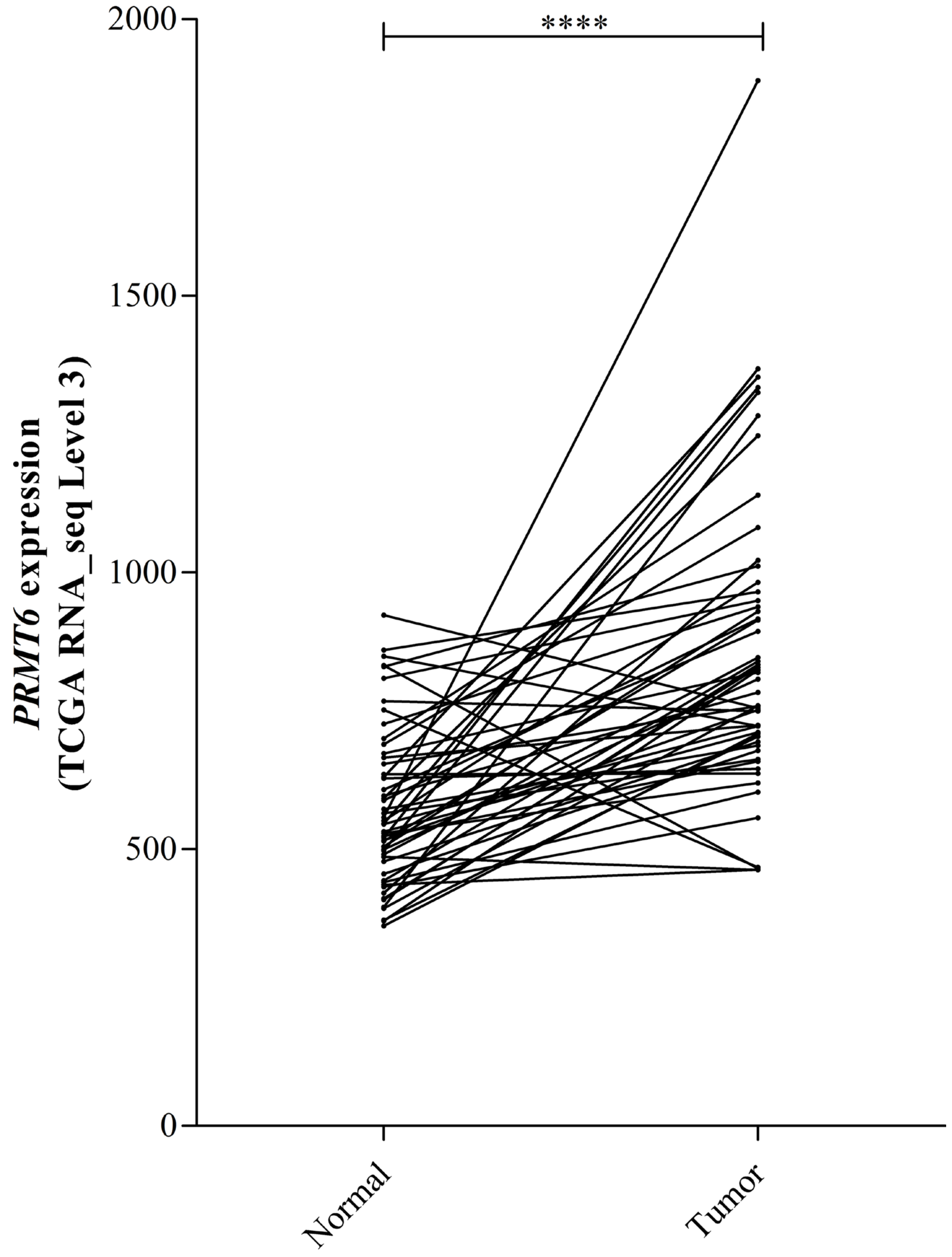

Supplementary Figure S1: PRMT6 mRNA expression is significantly increased in prostate cancer in patients from TCGA dataset (497 PCa vs. 52 normal tissue samples;**, p<0.0001) (A).** TCGA mRNAseq levels from 52 matched tumor and normal sample of prostate cancer patients from TCGA. (****, p<0.0001).

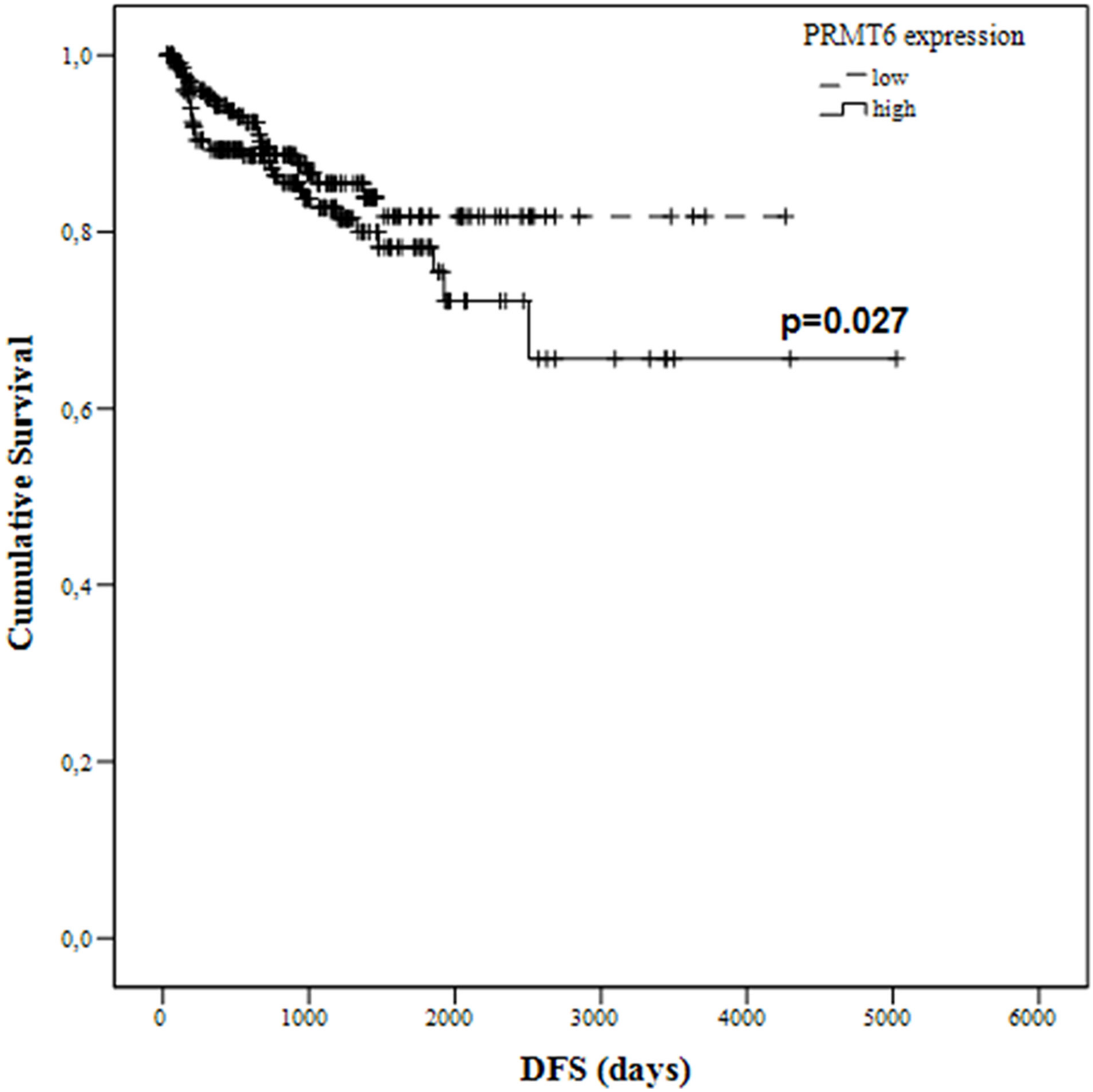

Supplementary Figure S2: Kaplan–Meier estimated disease-free survival curves for PCa patients. Disease-free survival curves of 428 PCa patients according to mRNA expression levels of PRMT6. The results presented were categorized according to median expression value.

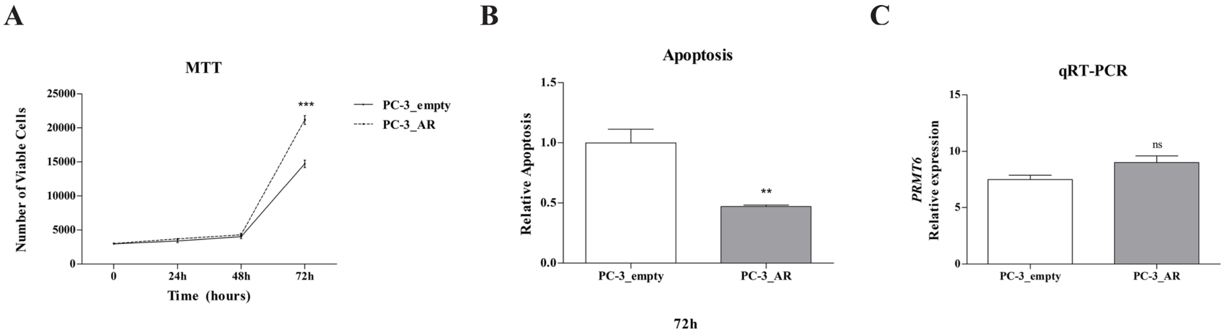

Supplementary Figure S3: Impact of AR induction in PC-3: A. cell viability by MTT assay at 0h, 24h, 48h and 72h; **B.** apoptosis by APOPercentage in Sh-Scramble at 72h in Sh-Scramble and Sh-PRMT6; **C.** Effect of AR ectopic expression in PRMT6 transcript levels in PC-3 cells. **p<0.01, ***p<0.001 (Mann-Whitney U-test).

Supplementary Table S1: TaqMan® Gene Expression Assays' references

Gene	Assay reference
AR	Hs00171172_m1
CD44	Hs01075861_m1
GUSB	Hs99999908_m1
MLL1	Hs00610538_m1
MLL2	Hs00231606_m1
MLL3	Hs01005521_m1
MLL4	Hs00207065_m1
MLL5	Hs00218773_m1
MMP9	Hs00234579_m1
MYC	Hs00153408_m1
PRMT6	Hs00250803_s1
SMYD3	Hs00224208_m1
